# Oxidative Storm Induced by Tryptophan Metabolites: Missing Link between Atherosclerosis and Chronic Kidney Disease

**DOI:** 10.1155/2020/6656033

**Published:** 2020-12-29

**Authors:** Iwona Kwiatkowska, Justyna M. Hermanowicz, Michal Mysliwiec, Dariusz Pawlak

**Affiliations:** ^1^Department of Pharmacodynamics, Medical University of Bialystok, Mickiewicza 2c, 15-222 Bialystok, Poland; ^2^Department of Clinical Pharmacy, Medical University of Bialystok, Mickiewicza 2c, 15-222 Bialystok, Poland; ^3^Ist Department Nephrology and Transplantation, Medical University, Bialystok, Zurawia 14, 15-540 Bialystok, Poland; ^4^Lomza State University of Applied Sciences, Akademicka 14, 18-400 Łomża, Poland; ^5^Department of Pharmacology and Toxicology, University of Warmia and Mazury in Olsztyn, Warszawska 30, 10-082 Olsztyn, Poland

## Abstract

Chronic kidney disease (CKD) occurrence is rising all over the world. Its presence is associated with an increased risk of premature death from cardiovascular disease (CVD). Several explanations of this link have been put forward. It is known that in renal failure, an array of metabolites cannot be excreted, and they accumulate in the organism. Among them, some are metabolites of tryptophan (TRP), such as indoxyl sulfate and kynurenine. Scientists have become interested in them in the context of inducing vascular damage in the course of chronic kidney impairment. Experimental evidence suggests the involvement of TRP metabolites in the progression of chronic kidney disease and atherosclerosis separately and point to oxidative stress generation as one of the main mechanisms that is responsible for worsening those states. Since it is known that blood levels of those metabolites increase significantly in renal failure and that they generate reactive oxygen species (ROS), which lead to endothelial injury, it is reasonable to suspect that products of TRP metabolism are the missing link in frequently occurring atherosclerosis in CKD patients. This review focuses on reports that shed a light on TRP metabolites as contributing factors to vascular damage in the progression of impaired kidney function.

## 1. Introduction

Chronic kidney disease (CKD) is one of the most commonly occurring diseases in the world, with more than 850 million people afflicted [1]. As a consequence of impaired kidney filtration, metabolites, which are normally excreted from the body, accumulate and can lead to systemic damage. They are generally called uremic toxins and according to the European Uremic Toxins Work Group (EUTox) database, 146 compounds are classified to this category [2]. Because of their different physicochemical features, they have been divided into three groups: small solutes, middle molecules, and protein-bound toxins. The latter are extremely doubtful, because dialysis, commonly used as a treatment option in CKD patients, is inefficient in their elimination. Therefore, they may be responsible for systemic damage in CKD patients, even in those who receive dialysis.

Atherosclerosis, a chronic inflammatory disease, is characterized by the accumulation of inflammatory cells and lipids in the artery wall, intima thickening, and vessel calcification. Over decades, it has afflicted more and more people, with increasing number of patients suffering from the cardiovascular disease. One medical condition that increases the risk of atherosclerosis is CKD. There is growing evidence that vascular endothelium damage begins in an early stage of CKD. The consequence is higher mortality from CVD in CKD patients than in patients without kidney impairment [3]. Several uremic toxins are considered ROS generators, and since it is known that their serum levels in CKD patients are elevated, efforts are being taken to establish their role in atherosclerosis development [4]. This review focuses on the link between renal impairment and atherosclerosis and on oxidative stress as a contributing factor to the development of these diseases separately and as a link between these two comorbid conditions. The main emphasis is on tryptophan (TRP) metabolites as potential therapeutic goals, the targeting of which could be beneficial in overcoming atherosclerosis incidence in CKD.

## 2. Oxidative Stress (OS) and Reactive Oxygen Species (ROS) in Chronic Kidney Disease and Atherosclerosis

Oxidative stress (OS) develops in conditions of imbalance between antioxidants and oxidants, with a predominance of the latter. Those highly reactive chemicals are represented by agents like superoxide (O_2_−), alkoxyl radical (RO·), peroxyl radical (ROO·), hydroxyl radicals (OH·), peroxynitrate (ONOO−), hydrogen peroxide (H_2_O_2_), ozone (O_3_), and hypochlorous acid (HOCl) [5]. Under physiological conditions, ROS play a role as second messengers and help to regulate processes like growth, signaling, apoptosis, and systemic functions, i.e., regulation of blood pressure or immune response [6]. Nevertheless, when produced in excess, they are harmful for cells, by causing lipid, protein, and DNA oxidation. That leads to systemic detrimental role of ROS, suggested in the pathogenesis of neurodegenerative disorders, cancer, diabetes, cardiovascular diseases, and kidney diseases [7–9]. On account of their physiological role in the body, there are multiple naturally occurring structures responsible for ROS generation. The main is the mitochondria, which, via electron chain transport, take part in ATP production. This process requires the company of nicotinamide adenine dinucleotide (NADH) and flavin adenine dinucleotide (FADH_2_). However, during the electrons' migration between complexes, some of them can escape in a process called “electron leaking” [10]. This leads to the formation of reactive species, such as superoxide (⁠O_2_^−^⁠) and hydrogen peroxide (H_2_O_2_). Another broad source of ROS is the NADPH oxidases (NOX) family, which contains seven members [11]. These enzymes are responsible for electron transportation from the cytosol to the extracellular space. In this location, they are coupled with molecular oxygen. Moreover, NOX4 can directly produce H_2_O_2_ by itself, which enhances its role in oxidative stress generation [12]. Other enzymes that can directly produce ROS agents are those belonging to the nitric oxide synthase (NOS) family. From three isoforms, endothelial NOS (eNOS), responsible for maintaining proper vascular tone, is essential in the pathogenesis of cardiovascular disease. ROS production takes place in a process called eNOS uncoupling, where an enzyme produces superoxide instead of NO [13]. Xanthine oxidase (XO) takes part in the conversion of hypoxanthine to uric acid, with results in O_2_·^−^ and H_2_O_2_ production [14]. It was proven that XO contributes to endothelial damage and the pathogenesis of CKD [15, 16]. Oxidative stress and ROS from different sources are suspected to be involved in the development of CKD and atherosclerosis. Moreover, they may be the missing link in the observed increased prevalence of atherosclerosis in CKD patients. Due to such a possibility, efforts are being made to discover in which mechanisms enhanced ROS production occurs in kidney impairment, which would explain a looping series of events: increased ROS level–kidney impairment–upregulated level of ROS–atherosclerosis. Thus far, accumulating tryptophan metabolites are the main suspects in this process.

During the development of CKD, several structural changes in the kidneys can be noticed. Increased apoptosis of podocytes, a reduction of functional nephrons, their sclerosis, and hypertrophy are hallmarks of kidney impairment [17]. Reactive oxygen species and oxidative stress are suspected to take part in this pathogenesis, and the results of experiments comparing ROS marker levels between healthy volunteers and CKD patients serve as support of this view. Elevated ROS levels are observed in the latter. Systemic ROS content needs to be evaluated by their markers, such as end products of lipid peroxidation, DNA damage, or the oxidation of proteins and amino acids, because of the unstable nature of ROS, which makes direct measurement impossible [18]. Aberration of ROS levels can be detected from the early stages of the disease and is noticeable in both adults and children [19, 20]. Also, experimental proofs of antioxidant intake in CKD patients suggest that molecules like vitamins C and E and omega-3 polyunsaturated fatty acids improve CKD patient conditions [21]. Reinforced efforts are made to explain how OS and ROS contribute to renal impairment. The kidneys, as large energy consumers, are rich in mitochondria, an organelle responsible for ATP production. As described above, mitochondria are one of the main sources of ROS, but they are also highly vulnerable to oxidative stress. Because of that, it is worth noting that mitochondria can be the cause and the victim of CKD, and a positive correlation between the stage of CKD and lowered mitochondrial DNA (mtDNA) was proven [22, 23]. It was shown that mitochondrial ROS can be generated after exposition to free fatty acids (FAAs), which have an influence on the loss of mitochondrial membrane potential [24]. This event leads to cytochrome c release, followed by the activation of the caspase cascade and finally podocyte apoptosis [25]. Palmitic acid was identified as one of the agents that cause the overproduction of mitochondrial superoxide in podocytes, followed by damage to those organelle and caspase-depended cell death in the final stage [26, 27]. In another study, podocyte injury was triggered by exposure to aldosterone, which enhances mitochondrial derived ROS production [28]. Podocyte apoptosis has been also induced by plasminogen (Plg) in the mechanism including ROS production by NADPH oxidase (NOX). Moreover, in the same experiment, the authors highlighted the crosstalk between Plg-activated NOX2 and the induction of mitochondrial NOX4 via the generation of O_2_^−^ by NOX2 [29]. The gathered data show that mitochondria-derived ROS take part in podocyte injury, which then leads to the progression of CKD. Dysfunction and apoptosis of podocytes are one of the features occurring in renal fibrosis. Another event that led to this state, and then to CKD progression, included increased fibroblast proliferation, the epithelial–mesenchymal transition (EMT), and the accumulation of the extracellular matrix (ECM) and mesangial cells as well as thickening of the tubular and glomerular membranes. Accumulation of proinflammatory cytokines and chemokines followed by chronic inflammation contributes to those negative changes [30]. Oxidative stress enhances fibrosis and CKD progression by making an impact on the aforementioned processes, and experimental studies indicate a significant decrease in renal fibrosis after OS decreasing [31]. Accumulation of ECM is induced by NOX-depended ROS generation, which promotes fibroblast transdifferentiation to myofibroblasts [32]. ROS-provoked upregulation of TGF-*β*1 activates the PI3K/Akt signaling pathway and thus epithelial to mesenchymal transition [33, 34]. All of these pathological changes lead to a worsening of kidney function and deterioration of filtration efficiency. As a result, metabolites accumulate and through different mechanisms affect the body's structures, causing further damage.

Atherosclerosis is characterized by endothelial dysfunctions, inflammatory processes, and enhanced lipoprotein storage, with consequent plaque formation. Oxidative stress and ROS are involved in atherosclerosis development. Recently, it was reported that monocytes express NOX5, which contribute to ROS formation, and oxLDL enhances its expression. Moreover, a comparison of an atherosclerotic and a nonatherosclerotic artery depicted significant upregulation of NOX5 in the first group [35]. Foam cell formation can also be enhanced by xanthine oxidase (XO), which as already mentioned is responsible for ROS generation. This naturally occurring protein in vascular smooth muscle cells stimulates the LOX-1 (lectin-like oxidized low-density lipoprotein receptor-1) expression, with subsequent oxLDL accumulation and proinflammatory mediator release. Moreover, by increasing arginase expression, oxLDL causes eNOS uncoupling, resulting in intensified ROS production and enhancing pathological process [36, 37]. ROS also contribute to cell apoptosis in a way dependent on the Fas ligand or by the activation of NF-*κ*B via the p38MAPK or PI3K pathways [38, 39]. Moreover, oxLDL is a source of proinflammatory agents, i.e., IL-12 and IL-18, which act like chemoattractants for T cells. They in turn secrete TNF-*α* and IFN-*γ*, which increase endothelial cell inflammation and apoptosis [40]. Additionally, according to results obtained by Ng et al., INF-*γ* leads to endothelial cell hyperpermeability by the activation of the p38MAPK kinase and actin rearrangement, which loops events leading to atherosclerosis development [41]. The same cytokine is involved in increasing plaque vulnerability by the inhibition of collagen production, thereby contributing to an increased risk of rupture. The aforementioned XO involved in the LOX-1 expression has been indicated as a receptor, involved in VSMC migration [42, 43]. These cells play a role in the different stages of atherosclerosis by secreting chemokines, monocyte attraction, and conversion to foam cells, which leads to their apoptosis and cholesterol release, thereby enlarging its generally accessible source. Moreover, they take part in fibrous cap formation and VSMC viability, which is essential in maintaining plaque stability. Those cells are also a source of calcifying microvesicles, and via these VSMCs participate in vessel calcification—another hallmark of atherosclerosis [44]. The above observations lead to the conclusion that reactive oxygen species are the starters that begin a cascade of events leading to atherosclerosis development.

### 2.1. Crosstalk between CKD and Atherosclerosis

It is well known that cardiovascular disease is one of the main causes of premature death in patients suffering from chronic kidney disease [45]. Observation of atheromatous plaques in CKD and non-CKD patients showed differences between them in terms of calcification level and increased the hydroxyapatite content in the first group. A study of end stage renal disease patients from 2018 showed that atherosclerosis developed in a majority of end stage renal disease (ESRD) patients [46]. Accelerated atherosclerosis development was observed also in research from the same year that compared atherosclerotic calcification in ESRD hemodialysis patients and healthy controls [47]. Kopel et al. reported greater impairment in vascular function in CKD patients than in the control group. Moreover, using the measure of nitroglycerine-mediated dilatation, they revealed that vascular dysfunction appeared in a mechanism dependent on smooth muscle cell malfunction [48]. In a study conducted by Pawlak et al., elevated OS levels and endothelial injury markers were revealed in hemodialysis patient with coexisting CVD, compared with control [49]. Blood samples derived from children with CKD were characterized by increased levels of endothelial microparticles, markers of endothelial dysfunction, when compared with healthy controls [50]. Similar results were obtained in research focusing on hemodialysis in children with end stage renal disease. Increased levels of TG, cholesterol, and LDL and decreased levels of HDL, which is a marker of premature atherosclerosis development, were detected in their blood samples [51]. Vorm et al. showed increased von Willebrand factor levels, another indicator of endothelium impairment in CKD and ESRD patients [52]. The gathered observation remains in line with the inference made by Gennip et al., who reported increased serum levels of endothelial dysfunction biomarkers in ESRD as compared with controls [53]. Altogether, these reports lead one to the conclusion that changes occurring in CKD are responsible for worsening the vascular condition and can lead to atherosclerosis development. Since traditional risk factors, such as hypertension or diabetes, are not sufficient to explain those pathological changes, new agents involved in those processes are widely sought after. Oxidative stress and reactive oxygen species are gaining more and more interest as nontraditional risk factors of CVD associated with CKD. Establishing their role as atherosclerosis mediators in CKD could be useful in developing a therapy to improve patient outcomes and extend their lifespan. Decreased efficacy of glomerular filtration with subsequent metabolite accumulation leads to the hypothesis that within those substances some of them have an impact on antioxidant compounds or ROS generation.

### 2.2. Indoxyl Sulfate, a Product of Tryptophan Degradation and Its Biological Activity

**Figure 1:**
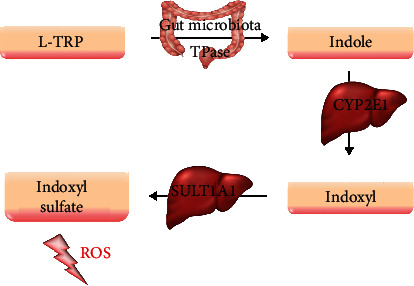
Tryptophan metabolism pathways, including a derivative involved in the production of reactive oxygen species. CYP2C1: cytochrome P450 2C1; L-TRP: L-tryptophan; ROS: reactive oxygen species; SULT1A1: sulfotransferase 1A1; TPase: tryptophanase.

**Figure 2:**
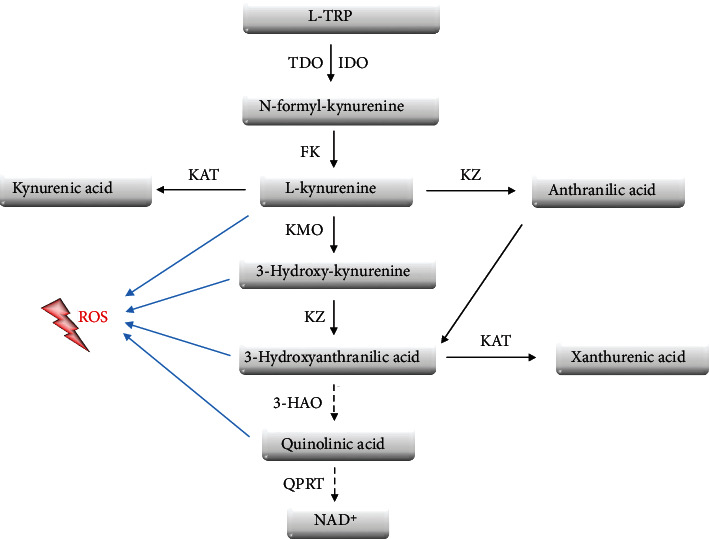
Tryptophan metabolism pathways, including derivatives involved in the production of reactive oxygen species. 3-HAO: 3-hydroxyanthranilate-3,4-dioxygenase; FK: formidase; IDO: indoleamine 2,3-dioxygenase; KAT: kynurenine aminotransferase; KMO: kynurenine 3-monooxygenase; KZ: kynureninase; L-TRP: L-tryptophan; QPRT: quinolinic acid phosphoribosyltransferase; ROS: reactive oxygen species; TDO: tryptophan dioxygenase.

Tryptophan is an essential amino acid, which needs to be supplied with nourishment. It is metabolized via three major catabolic pathways: the indole pathway (Figure 1), the kynurenine pathway (Figure 2), and the serotonin pathway [54].

TRP to indole conversion depends on the host microorganism and takes place in the intestine. After this step, indole undergoes metabolic changes in hepatocytes with indoxyl sulfate (IS) production [55]. This agent is one of the best described uremic toxins.

IS is a small molecule that in at least 90% binds to plasma proteins. The remaining free fraction contributes to numerous pathological conditions, including the induction of oxidative stress and inflammation [56]. By NF-*κ*B p65 phosphorylation, IS leads to an increase of p21 and p53 expression. Concomitantly, release of TGF-*β*1, a monocyte chemoattractant protein-1, ET-1, and osteopontin, which increase the biological activity of TGF-*β* manifested by the stimulation of metallopeptidase inhibitor-1 and collagen biosynthesis, is observed [57]. These changes are often accompanied by systemic disturbances, such as cardiovascular disorders, cardiac fibrosis, arterial calcification, osteodystrophy, and kidney tissue damage [58–60]. Several of the biological effects exerted by the free fraction of IS occur via the activation of the aryl hydrocarbon receptor (AhR). This interaction impairs vascular structures through inhibiting endothelial cell proliferation and decreasing DNA synthesis in those cells [61]. Moreover, it enhances the expression of monocyte chemoattractant protein-1, which is involved in atherosclerosis development [62]. Additionally, AhR activation by IS leads to the AhR-NF-*κ*B/MAPK cascade activation followed by the induction of inflammation [63]. The IS-AhR interaction also impairs the skeletal system. Liu et al. indicated the IS/AhR/MAPK signaling pathway as a mediator of impaired osteoblastogenesis [64]. Another experiment revealed that the effect of IS on osteoclastogenesis depends on the concentration and exposure time and that osteoclast differentiation decreases under the chronic impact of IS. It was confirmed that the observed effect occurs through AhR activation [65]. In the central nervous system, IS activates AhR in astrocytes, which leads to increased ROS production [66]. Moreover, experimental outcomes indicate that this interaction leads to inflammation in primary astrocytes and mixed glial cells [67].

Due to the systemic toxicity of IS, methods of its elimination in patients with impaired renal function are in constant development. The high degree of binding with albumins makes hemodialysis an inefficient means of removing IS from the blood. For this reason, different ways of increasing the effectiveness of blood purification are being contemplated. One possibility is the use of binding competitors that displace IS from albumin binding, thus augmenting its free form, which can be dialyzed. The effectiveness of salvianolic acids as a factor that enhances IS removal from blood was examined in an experimental animal model [68]. Another binding competitor that can be used to increase IS elimination is ibuprofen. Its arterial infusion during dialysis treatment significantly decreased the remaining IS amount in serum [69]. Ibuprofen increased the free fraction of IS approximately three-fold in uremic plasma, higher than the free fraction generated by furosemide. Moreover, the addition of furosemide to ibuprofen intensified its displacement effect [70]. In turn, Shi et al. proved both an increase in the free form of IS in rat plasma and higher reduction ratio in a group treated with intravenous lipid emulsion (ILE) than in the control [71]. Free fatty acids, which are the components of ILE that have a high affinity for albumins, so they also act as binding competitors. Another approach in increasing the efficacy of hemodialysis is based on using an adsorbent that binds IS. The addition of poly-cyclodextrin, which binds IS via hydrophobic interactions and hydrogen bonds, to dialysate of the exterior dialyzer resulted in a significant increase in uremic toxin removal from plasma [72]. Yamaguchi et al. demonstrated that the administration of oral adsorbent AST-120 also decreased IS plasma content [73]. An alternative way of improving the efficacy of hemodialysis is modifying the dialysis fluid through the replacement of buffer solution. Hyšpler et al. indicated that use of acetate buffer predominates over the citrate one in terms of enhancing IS elimination [74].

The aspect that cannot be omitted in the attempts of decreasing IS levels is the fact that it is a metabolite of an exogenous amino acid. Thus, diet modifications may be crucial in IS management of CKD patients. It was proved that a very low protein diet (VLPD) helps reduce total and free IS in patients with renal impairment [75]. Comparison of a low protein diet with a very low protein diet showed differences in the efficiency, with a predominance of the latter, indicating that protein content is a key factor in decreasing plasma IS levels [76]. Altogether, these data indicate that there is no unique perfect method that could be used as an effective way to sufficiently eliminate accumulated IS in CKD patients, and for this reason, new approaches need to be found.

### 2.3. Indoxyl Sulfate in Chronic Kidney Disease

**Figure 3:**
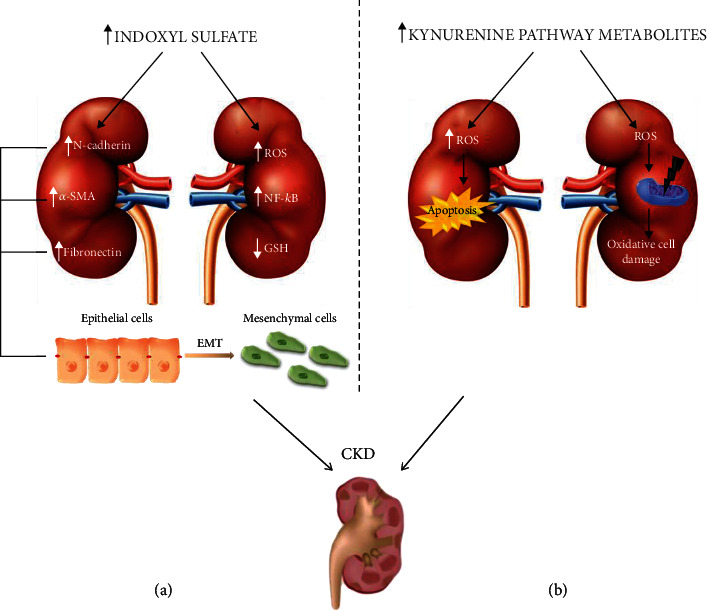
Contribution of tryptophan metabolites, indoxyl sulfate (a) and kynurenines (b), in the development of chronic kidney disease, including reactive oxygen species as a damaging factor. CKD: chronic kidney disease; EMT: epithelial mesenchymal transition; NF-*κ*B: nuclear factor kappa-light-chain-enhancer of activated B cells; ROS: reactive oxygen species.

Cumulation of IS in kidney impairment was confirmed by Yeh et al., who indicated its increased serum levels in CKD patients when compared to healthy volunteers [77]. Moreover, it was reported that along with CKD advancement serum IS levels increase, which emphasizes reduced IS excretion together with progressive renal failure [78]. A meta-analysis from 2015 showed an association between elevated levels of free form IS and increased mortality in CKD patients, and comparison of clinical outcomes of hemodialysis patients revealed an elevated risk of all-cause mortality in those who had higher serum IS levels [79, 80]. These facts lead one to consider that IS not only accumulates under kidney impairment but also enhances its progression by damaging renal structures, thus confirming its toxic character (Figure 3(a)). Ellis et al. showed increased expression of proapoptotic protein in proximal tubular epithelial cells (PTECs) and human renal tubular epithelial cells (HK-2) under the impact of IS. Additionally, in the same conditions, they indicated increased hypertrophy and expression of profibrotic molecules, well known hallmarks of CKD, in tested cells [81]. An *in vitro* study of HK-2 cells reveled increased expression of *α*-smooth muscle actin (*α*-SMA), N-cadherin, and fibronectin—markers of epithelial-mesenchymal transition, which is another pathological process leading to CKD progression [82]. Enhanced EMT was observed as well in rat renal tubular epithelial cells (NRK-52E) after IS stimulation, thereby confirming its involvement in disease development [83]. This leads to the question as to which IS mechanism causes its toxic effects. The available data show that the administration of an antioxidant and NADPH inhibitor attenuates the proinflammatory effects exerted by IS in proximal tubular cells [84, 85]. Wang et al. discovered that indoxyl sulfate induces oxidative stress, upregulates of NF-*κ*B with following increase in the CYP24 expression in renal tubule epithelial cells (Figure 3(a)) [85, 86]. That remains in accordance with a previous observation that IS enhances ROS production and activates NF-*κ*B in HK-2 cells and rat models, confirming the role of oxidative stress in the toxic mechanism of the described molecule [87]. Further intensified energy consumption, changes in mitochondrial membrane potential and IV complex activity, with consequent reduction of mitochondrial mass on the HK-2 cell line and impairment of mitochondrial functions in nephrectomized mice was demonstrated by Sun and colleagues. According to the authors' conclusions, IS enhances mitochondrial oxidative stress in HK-2 cells, which contributes to cellular damage present in CKD progression [88]. The key role of oxidative stress in the damaging mechanism of IS was also confirmed by Edamatsu. The obtained results found a decrease in total glutathione levels in porcine renal tubular cells and their higher vulnerability to oxidative stress (OS) with enhanced apoptosis after the administration of mixed uremic toxins containing IS [89]. The above data indicate that IS is an important mediator of ROS production in renal structures.

### 2.4. Indoxyl Sulfate in Atherosclerosis

**Figure 4:**
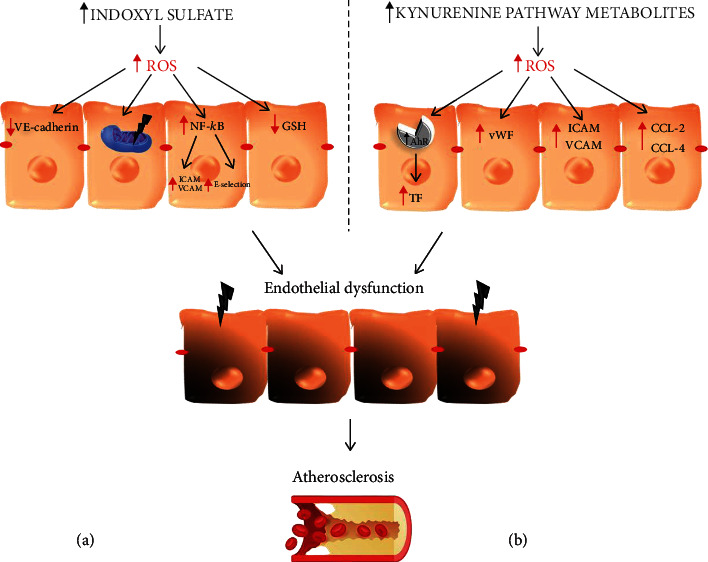
Contribution of tryptophan metabolites, indoxyl sulfate (a) and kynurenines (b), in the development of atherosclerosis, including reactive oxygen species as a damaging factor. AhR: aryl hydrocarbon receptor; CCL-2, -4: macrophage inflammatory protein 2, 4; GSH: glutathione; ICAM-1: intercellular adhesion molecule; NF-*κ*B: nuclear factor kappa-light-chain-enhancer of activated B cells; ROS: reactive oxygen species; VCAM-1: vascular cell adhesion molecule-1; VE-cadherin: vascular endothelial cadherin; vWF: von Willebrand factor.

Observations of IS dealing with ROS generation lead one to assume that this uremic toxin contributes to vascular injuries via a similar mechanism, especially considering the endothelium is highly vulnerable to oxidative stress. The existing data serve as evidence that IS has an effect on endothelial cell viability, permeability, activation, and calcification. Namely, it is involved in multiple steps leading to atherosclerosis (Figure 4(a)). Limited viability of human umbilical vein endothelial cells (HUVECs) under the influence of IS was observed by Li and colleagues [90]. That remains in line with outcomes obtained by Lee et al., who reported not only decreased HUVEC viability after IS treatment but also detected parallels between higher dosage of IS and escalating ROS generation. Moreover, research identified impaired mitochondrial function, reduction in mtDNA copy number, and their decreased mass in IS-treated HUVECs, when compared to untreated controls. These effects have been reversed after the administration of antioxidants such as vitamin C or NAC, which indicates oxidative stress, derived from mitochondria, involvement in the toxic action of IS [91]. Searching of other sources of ROS in endothelial cells after IS exposure led to outcomes indicating enhanced NADPH oxidase activity with simultaneous decreasing eNOS activity and inhibition of NO production in human aortic endothelial cells [92]. Dou et al., who additionally proved a negative impact on glutathione levels in HUVECs exposed to IS, also indicated an NADPH oxidase-depended mechanism [93]. Shen et al., who reported enhanced activity of NADPH oxidase in IS-treated HUVECs, with subsequent intensification of E-selectin expression, obtained similar results. They also found that IS activates NF-*κ*B, which leads to the same effect on the abovementioned protein. Thus, the authors discuss the existing cascade, activation of NADPH oxidase–ROS generation–activation of NF-*κ*B–upregulation of E-selectin expression, as a mechanism through which IS triggers its effect [94]. The activation of NF-*κ*B by IS was also reported by Tumur et al., who indicated that NF-*κ*B enhances the expression of ICAM-1 in HUVECs and that the administration of an antioxidant (NAC) attenuates this effect [95]. That strengthens the hypothesis of oxidative stress involvement in mediating IS toxicity toward endothelial cells. Enhanced ICAM-1 and VCAM-1 expression, thus endothelial activation involved in atherosclerosis, was reported after acute and chronic distribution of IS by Six and colleagues [96]. Those results remain in line with outcomes obtained by Lu et al., who documented morphological changes and HUVEC degeneration after IS treatment [97]. The same authors point to increased intracellular ROS production and decreased eNOS and VE-cadherin expression after IS stimulation. Altogether, these data highlight the role of oxidative stress as a mediator of the IS toxic effect exerted toward vascular structures.

### 2.5. Kynurenine and Its Biological Activity

TRP undergoes extensive metabolism along several pathways, of which kynurenine is one of the most significant and occurs in the highest level (Figure 2) [98]. To date, three enzymes have been identified to take part in the first step of TRP conversion—two isozymes of indoleamine 2,3-dioxygenase (IDO1, IDO2) and tryptophan dioxygenase (TDO) [99]. All of them take part in the oxidative degradation of the aromatic ring with kynurenine production, and differences between them were observed in catalytic activity [100]. The newly created kynurenine is further converted into active metabolites, 3-hydroxykynurenine (3-HK), kynurenic acid (KYNA), and hydroxyanthranilic acid (3-HAA), together called kynurenines [101]. Kynurenines gained researchers' interest due to alternations in their metabolic pathway and level in disparate medical conditions, such as schizophrenia, Alzheimer's disease, Parkinson's disease, different types of cancer, diabetes mellitus, cardiovascular disorders, or chronic kidney disease [102–110]. Metabolites of the kynurenine pathway exert diverse, sometimes opposite, roles on many biological processes, including inflammation, redox homeostasis, gluconeogenesis, and apoptosis [111]. Their accumulation may affect numerous cellular signaling pathways through AhR activation, leading to the disruption of the homeostasis of various organs. AhR is a ligand-activated transcription factor and has recently been highlighted as playing a critical role in the maintenance of cellular homeostasis. Therefore, its overactivation by the higher concentration of KYN and its metabolites may enhance cell aging processes and their death rate. Moreover, numerous KYN derivatives demonstrate toxic properties related to their ability to induce oxidative stress or the formation of excitotoxic complexes with insulin [111–114]. These alternations may manifest clinically in the form of systemic disorders, such as osteodystrophy, insulin resistance, neurological disorders, changes in blood pressure, anemia, hypercoagulability, atherosclerosis, and kidney tissue damage.

The effectiveness of hemodialysis for kynurenines elimination is limited. It has been reported that they are present in the dialysate, and their plasma level in hemodialyzed patients was higher compared with healthy volunteers [115]. Moreover, Pawlak et al. reported that metabolites of the kynurenine pathway accumulate in hemodialyzed patients [115]. Comparison of three renal replacement therapies (hemodialysis peritoneal dialysis, kidney transplantation) showed a significant increase of IDO activity in all three groups, when compared with the control group under peritoneal dialysis characterized by a meaningful increase of kynurenine levels when compared to others [116]. This indicates the inability of renal replacement therapy to eliminate KYN metabolites. The aspect that needs to be taken under the consideration is the fact that KYN content does not grow proportionally to the decrease in the GFR, which may indicate another mechanism involved in its elimination [111].

### 2.6. Kynurenines in Chronic Kidney Disease

The metabolites of the kynurenine pathway play a significant role in the modulation of physiological, as well as pathological processes, including redox homeostasis. KYN, the first product of TRP degradation, exerts prooxidant effects, and the aerobic irradiation of KYN produces superoxide radicals and leads to cytochrome C reduction [112]. Moreover, increased levels of KYN result in cell death through the ROS pathway in natural killer (NK) cells [117]. Accumulated KYN metabolites, via the ability to induce/potentiate oxidative stress, show a negative or toxic effect on many cellular processes, which may lead to cell damage, an increased rate of apoptosis, or triggering the inflammatory processes that reflect a disturbance of homeostasis of various organs and systems [98, 111, 112, 118, 119]. The impact of kynurenine metabolites on body homeostasis cannot be forgotten in the course of diseases where significant changes in their activity are observed [112, 120]. Alterations in the kynurenine system have been linked to CKD and atherosclerosis. The inflammatory process observed in the course of CKD causes an increase in IDO activity. Since IDO is involved in tryptophan metabolism, its induction leads to a decrease in its tissue and plasma concentration with a simultaneous increase in kynurenine pathway metabolite synthesis [111, 114, 120, 121]. In addition, a permanent reduction in the glomerular filtration rate observed in CKD contributes to the increasing level of KYN metabolites in the plasma and tissues. Pawlak et al. reported their accumulation both in experimental models of CKD and in uremic patients. In patients with uremia, the concentrations of KYN, KYNA, and QA were increased by 37-105%, 84-428%, and 394-1018% of the control values, respectively. These changes were accompanied by a significant increase in KYNA/KYN and QA/KYN ratios, reflecting the increased activity of kynurenine pathway enzymes [115, 122, 123]. A positive correlation between the level of KYN metabolites, examined in patients' blood or animal tissues and the degree of renal insufficiency, was previously confirmed [115, 122, 124, 125]. Moreover, KYN metabolites, particularly 3-hydroxykynurenine, are associated with oxidative stress in ESRD patients. The same authors also demonstrated strong positive associations between KYNA, QA, and SOX markers in uremic syndrome. The values of KYN, QA, the QA/KYN ratio, total peroxide, Cu/Zn superoxide dismutase, and malondialdehyde were significantly higher than in healthy people [126]. The biological activity of QA is mainly related to the agonistic action at N-methyl-D-aspartate (NMDA) receptors, which are located both in the central nervous system and in many cells on the periphery [111]. NMDA receptors and free radical processes are involved in the excitotoxic mechanism of QA activity, mainly related to the accumulation of peroxynitrite (ONOO-) in the cell, an increase in nitric oxide synthase activity, a decrease in superoxide dismutase activity, and intensification of the lipid peroxidation process. Increased NO synthesis causes a cellular energy deficit dependent on the reaction of the peroxynitrite (ONOO-) molecule with the enzymes of the tricarboxylic acid cycle, mitochondrial respiratory chain, mitochondrial calcium metabolism, or by DNA damage [127].

As mentioned above, the accumulation of KYN metabolites in the course of CKD may induce oxidative cell damage, which leads to inflammatory processes. Okuda et al. reported that the excess of 3-HKYN enhances ROS generation, leading to mitochondria function impairment [128]. It also dysregulates the respiratory chain parameters, reduces the respiratory control index, decreases the adenosine diphosphate/oxygen and glutamate/malate ratio in mitochondria, and uncouples the respiratory chain and oxidative phosphorylation [128, 129]. These alternations are probably due to the increase in ROS concentration associated with the KYN intensified auto-oxidation process as a result of its accumulation in CKD. Moreover, their ability to produce ROS through increased oxidative stress level severity in renal cells leads to exacerbated cell damage and accelerated rate of apoptosis in renal tissues during CKD progression (Figure 3(b)) [111, 129, 130]. The above data indicate that the accumulated KYN metabolites may participate in the development of kidney cell dysfunction leading to their damage resulting in organ failure. Therefore, the inhibition of TRP metabolic pathway activity, thanks to a slow or even stop in the destructive processes in the kidney, could be a potential therapeutic goal in uremic patients.

### 2.7. Kynurenines in Atherosclerosis

The available data demonstrated that the accumulation of toxic TRP metabolites in the body seems to be an important factor affecting vascular endothelial dysfunction leading to atherosclerosis (Figure 4(b)) [110, 112]. It has been reported that the KYN/TRP ratio is associated with carotid intima-media thickness, a presymptomatic predictor of atherosclerosis [131, 132]. Elevated levels of 3-HKYN have also been documented in patients with cardiovascular diseases. In addition, the accumulation of QA was independently related with the progression of atherosclerosis in the plasma of uremic patients [126]. The long-term accumulation and biological properties of KYN metabolites negatively affected the parameters of the erythrocytic system in patients [133]. KYN and its metabolites were also associated with hyperfibrinolysis. Kaminski et al. reported that AA is negatively correlated with the tissue plasminogen activator during severe-to-end-stage CKD [134]. Disturbances of the fibrinolytic system cause an increase in the prothrombotic potential, responsible for the pathogenesis of atherosclerosis and cardiovascular disorders. Wang et al. provided evidence that the overactivation of the kynurenine pathway was associated with increased oxidative stress and inflammation [112]. It was proven that the activation of the aryl hydrocarbon receptor AhR and also the promotion of oxidative stress by KYN, 3-KYN, 3-HAA, and QA may be important players in the initiation and progression of atherosclerosis [119, 124, 126, 135]. Pawlak et al. documented that KYN and 3-HKYN were positively associated with inflammation and SOX markers in uremic syndrome. They concluded that a link between kynurenine pathway activation and increased oxidative stress, inflammation, and the progression of atherosclerosis exists [133]. Watanabe et al. showed that overexpression of indoleamine 2,3-dioxygenase in coronary atherosclerotic plaques via increased oxidative stress level, and the AhR pathway stimulation enhanced tissue factor expression (TF) in activated macrophages [136]. The induction of oxidative stress and AhR activation by KYN and its metabolites also increased inflammation [131–133]. Inflammation is crucial to atherosclerosis, since it contributes to coronary plaque instability, increased vulnerability to rupture or erosion, leading to thrombosis and myocardial infarction (MI) [137]. A positive correlation between KYN, 3-HKYN, AA, and QA with crucial factors associated with the development of atherosclerosis such as TF, von Willebrand factor (vWF), thrombomodulin, and prothrombin fragments F(1+2) concentration as well as sICAM-1 (soluble intercellular adhesion molecule-1) and sVCAM-1 (soluble circulating vascular cell adhesion molecule-1) level was noticed [114, 133, 138, 139]. KYN was also independently and significantly associated with elevated sICAM-1, whereas 3-hydroxyanthranilic acid was positively correlated with the concentration of CCL-2 and CCL-4 chemokines. Additionally, IDO plays a proinflammatory role in human diseases. It has been shown that it was recognized as a novel marker of immune activation in the early stages of atherosclerosis [110]. Its downstream metabolites induce overexpression of proinflammatory factors [140]. Kynurenine 3-monooxygenase has also been documented as another crucial regulator of inflammation [141]. The abovementioned relationship may represent one of the mechanisms involved in the development of atherosclerosis [141]. These data confirm that the aggressive nature of kynurenines may contribute to the induction and progression of atherosclerosis; it has a proinflammatory and prooxidative effect and causes endothelial dysfunction [142, 143]. It is worth mentioning that not all metabolites of the kynurenine pathway have a proatherogenic effect. It has been shown that KYNA concentration and the KYNA/KYN ratio were significantly lower in patients without cardiovascular disease, and they were positively associated with homocysteine levels [144, 145]. The KYNA mechanism of action seems to be related with the inhibition of homocysteine-induced cytotoxicity [144]. Moreover, increased KYNA in plasma concentration caused a decrease in triglyceride and cholesterol levels and an inhibition of the uptake of oxidized low-density lipoproteins by macrophages, which resulted in an inhibition of atherogenesis in a murine model [146, 147].

### 2.8. Changes of TRP Metabolism in the Course of CKD

As mentioned above, under normal conditions, TRP is metabolized mostly in the liver. Nevertheless, during pathological processes, like chronic kidney disease, its conversion changes significantly. Depletion of TRP in CKD patient serum has been reported multiple times [148, 149]. Moreover, the kynurenine to tryptophan ratio is elevated in those patients, which indicates the overactivity of enzymes that take part in TRP catabolism [150]. The increased activity of IDO1 is due to elevated IFN-*γ* levels, the main inductor of this protein. Also, increased cytokine levels can affect the hypothalamo-pituitary-adrenal axis, which in turn enhances TDO activity [150]. Altogether, those modifications of TRP metabolism lead to increased levels of its derivatives. This is consistent with scientific reports that indicate a correlation between increased KYN, 3-HKYN, XA, KYNA, AA, and QA and decreased GFR. The kidneys contribute to those changes in two ways. This organ is characterized by high expression of IDO, enhancing tryptophan depletion. Moreover, during kidney dysfunction development, excretion of TRP metabolites is insufficient, which leads to their accumulation. In the context of cardiovascular disorder occurrence in the progress of CKD, it is worth noting that alternation in TRP metabolism varies among groups with varied risk of CVD development. Konje et al. indicated a correlation between decreased basal levels of TRP and increased risk of CVD incident, which could suggest that changes in TRP metabolism are factors contributing to cardiovascular complications occurring in renal impairment [151].

### 2.9. TRP Pathway Metabolites—a Link between CKD and Atherosclerosis

The abovementioned experimental outcomes indicate IS and kynurenines as mediators of ROS generation, taking part in enhancing both CKD and atherosclerosis. Since it is known that atherosclerosis develops often in patients suffering from CKD, it is logical to suspect TRP metabolites as agents that by accumulating in kidney failure lead to vascular impairment. In nephrectomized rats, augmentation of aortic media thickness and decrease of the aortic lumen diameter was observed. The authors reported higher endothelial dysfunction, reduction of the eNOS expression, and diminution of NO production in nephrectomized rats on additional IS treatment when compared to controls. In this study, the authors confirmed ROS involvement in the IS mechanism of action, by showing a significant elevation of superoxide levels in the aortic walls of rats with kidney impairment [152]. Analysis of samples derived from CKD children also confirms the association between CKD severity, increased IS, and carotid artery intima-media thickness. Further, a correlation between this uremic toxin and marker of endothelial dysfunction persisted for 12 months, separately from other risk factors [153]. Similar results were obtained in uremic patients in whom KYN, QA, and the QA/KYN ratio were positively associated with IMT values (intima-media thickness). The authors observed that kynurenine pathway metabolites, via propagation of oxidative stress, are responsible for both endothelial dysfunction and IMT values in patients with chronic kidney disease [126]. Claro et al. evaluated the connection between kidney impairment and vascular response in CKD patients. Their outcomes indicate a relation between elevated IS levels, markers of vascular inflammation, and endothelial dysfunction, such as sFAS, sVCAM-1, and MCP-1, in those patients [154]. An experiment on mice with induced CKD showed that IS causes loss of endothelial cells and enhances the expression of adhesive molecules (ICAM-1, VCAM-1) [96]. Kynurenines were also positively associated with vWF, TM, sICAM-1, and sVCAM-1, which have been implicated as markers of endothelial cell dysfunction and intima-media thickness in the early stage of systemic atherosclerosis in the course of CKD [110]. The study conducted by Kamiński et al. shows a positive correlation between high levels of IS in CKD patients and TM, a marker of endothelium functionality. Moreover, the parallel between increased IS content and markers of oxidative stress, Cu/Zn SOD and H_2_O_2_, has been pointed out, which proves the predicted mechanism of action [155]. Another support of the thesis that IS links these two diseases comes from a study on predialysis CKD patients. Administration of oral IS adsorbent (AST-120) significantly improved flow-mediated dilation after diminution of serum IS levels. In this experiment, the authors indicated an NADPH-depended mechanism in ROS generation, which contributes to endothelial impairment mediated by IS [156].

Hypertension, a constant symptom of CKD, appears to have a complex association with endothelial dysfunction. In turn, hyperkynureninemia increases the risk of hypertension occurrence in patients with chronic kidney disease (CKD) [157]. In addition, Martinsons et al. pointed out that hyperkynureninemia may lead to the progression of cardiovascular disease, including hypertension, a known factor in the development of atherosclerosis [158, 159]. The above information serves as an argument weighted in favor of TRP metabolites as agents enhancing oxidative stress, which increases the risk of atherosclerosis in the progression of chronic kidney disease. These observations could help develop therapies targeting components of the tryptophan-kynurenine pathway in CKD to prevent cardiovascular consequences and improve patient outcomes.

## 3. Conclusion

The available data provide evidence that the accumulation of TRP toxic metabolites in the body seems to be one of the key factors underlying the development of uremic symptoms, including impaired lipid metabolism and vascular endothelial dysfunction, leading to atherosclerosis in the course of CKD. The main reason is their ability to induce oxidative stress, leading to the exacerbation of simmering inflammation. Complete understanding of the impact of TRP active metabolites on overall body homeostasis, as well as on the condition of many organs, may provide the basis for the development of innovative therapeutic options that improve the diagnosis and treatment of CKD and systemic disorders related with its progression, such as atherosclerosis.

## Abbreviations

3-HAA: Hydroxyanthranilic acid
3-HK: 3-Hydroxykynurenine
AhR: Aryl hydrocarbon receptor
Akt: Protein kinase B
ATP: Adenosine triphosphate
CCL-2, -4: Macrophage inflammatory protein 2, 4
CKD: Chronic kidney disease
CVD: Cardiovascular disease
DNA: Deoxyribonucleic acid
ECM: Extracellular matrix
EMT: Epithelial mesenchymal transition
eNOS: Endothelial nitric oxide synthase
ESRD: End stage renal disease
EUTox: European Uremic Toxins Work Group
FAAs: Free fatty acids
FADH2: Flavin adenine dinucleotide
GFR: Glomerular filtration rate
GSH: Glutathione
HDL: High-density lipoproteins
HK-2: Human renal tubular epithelial cells
HUVECs: Human umbilical vein endothelial cells
ICAM-1: Intercellular adhesion molecule
IDO1, IDO2: Indoleamine 2,3-dioxygenase type 1,2
IFN-*γ*: Interferon gamma
IL-12, 18: Interleukin 12, 18
IMT: Intima-media thickness
IS: Indoxyl sulfate
KYNA: Kynurenic acid
LDL: Low-density lipoproteins
LOX-1: Lectin-like oxidized low-density lipoprotein receptor-1
MCP-1: Monocyte chemoattractant protein-1
NAC: Acetylcysteine
NADH: Nicotinamide adenine dinucleotide
NADPH: Nicotinamide adenine dinucleotide phosphate
NF-*κ*B: Nuclear factor kappa-light-chain-enhancer of activated B cells
NK: Natural killer
NO: Nitric oxide
NOS: Nitric oxide synthase
NOX: NADPH oxidases
NRK-52E: Rat renal tubular epithelial cells
OS: Oxidative stress
oxLDL: Oxidized low-density lipoproteins
p38MAPK: p38 mitogen-activated protein kinases
PI3K: Phosphoinositide 3-kinases
Plg: Plasminogen
PTECs: Proximal tubular epithelial cells
QA: Quinolinic acid
ROS: Reactive oxygen species
sICAM-1: Soluble intercellular adhesion molecule-1
SMC: Smooth muscle cells
SOX: Increased oxidative stress
VCAM-1: Vascular cell adhesion molecule-1
TDO: Tryptophan dioxygenase
TF: Tissue factor
TGF-*β*1: Transforming growth factor *β*
TM: Thrombomodulin
TNF-*α*: Tumor necrosis factor alpha
TRP: Tryptophan
VCAM-1: Vascular cell adhesion molecule-1
VE-cadherin: Vascular endothelial cadherin
VSMCs: Vascular smooth muscle cells
vWF: von Willebrand factor
XO: Xanthine oxidase
*α*-SMA: Alpha smooth muscle actin.
